# Nano Meets Micro-Translational Nanotechnology in Medicine: Nano-Based Applications for Early Tumor Detection and Therapy

**DOI:** 10.3390/nano10020383

**Published:** 2020-02-22

**Authors:** Svenja Siemer, Désirée Wünsch, Aya Khamis, Qiang Lu, Arnaud Scherberich, Miriam Filippi, Marie Pierre Krafft, Jan Hagemann, Carsten Weiss, Guo-Bin Ding, Roland H. Stauber, Alena Gribko

**Affiliations:** 1 Nanobiomedicine Department, University Medical Center Mainz/ENT, Langenbeckstrasse 1, 55131 Mainz, Germany; 2 Laboratory of Tissue Engineering, Universitätspital Basel, Hebelstrasse 20, CH-4031 Basel, Switzerlandmiriam.filippi@usb.ch (M.F.); 3 Institut Charles Sadron (CNRS), University of Strasbourg, 23 rue du Loess, 67034 Strasbourg Cedex, France; 4 Institute of Biological and Chemical Systems-Biological Information Processing (IBCS-BIP), Postfach 3640, 76021 Karlsruhe, Germany; 5 Institute for Biotechnology, Shanxi University, No. 92 Wucheng Road, 030006 Taiyuan, China

**Keywords:** biocompatibility, circulating tumor cells, metastasis, microbubbles, nanomedicine, nanotechnology

## Abstract

Nanomaterials have great potential for the prevention and treatment of cancer. Circulating tumor cells (CTCs) are cancer cells of solid tumor origin entering the peripheral blood after detachment from a primary tumor. The occurrence and circulation of CTCs are accepted as a prerequisite for the formation of metastases, which is the major cause of cancer-associated deaths. Due to their clinical significance CTCs are intensively discussed to be used as liquid biopsy for early diagnosis and prognosis of cancer. However, there are substantial challenges for the clinical use of CTCs based on their extreme rarity and heterogeneous biology. Therefore, methods for effective isolation and detection of CTCs are urgently needed. With the rapid development of nanotechnology and its wide applications in the biomedical field, researchers have designed various nano-sized systems with the capability of CTCs detection, isolation, and CTCs-targeted cancer therapy. In the present review, we summarize the underlying mechanisms of CTC-associated tumor metastasis, and give detailed information about the unique properties of CTCs that can be harnessed for their effective analytical detection and enrichment. Furthermore, we want to give an overview of representative nano-systems for CTC isolation, and highlight recent achievements in microfluidics and lab-on-a-chip technologies. We also emphasize the recent advances in nano-based CTCs-targeted cancer therapy. We conclude by critically discussing recent CTC-based nano-systems with high therapeutic and diagnostic potential as well as their biocompatibility as a practical example of applied nanotechnology.

## 1. Introduction

**Figure 1:**
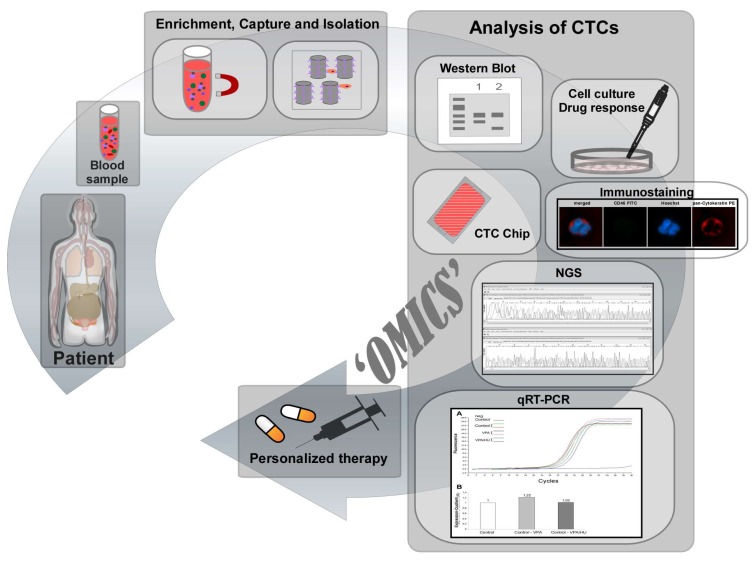
Overview of CTCs analysis of patients-derived CTCs including drug resistance detection and personalized therapy: Patients’ blood sample is screened and potential CTCs are captured and isolated by different isolation methods. Potential CTCs can be determined and used for further analysis to develop personalized medicine.

The application of engineered nanomaterials (NMs) in technical products is steadily growing in biotechnology and biomedicine [1]. Nanomedicine, i.e., the medical application of nanotechnology, is expected to play a vital role in early tumor detection and cancer treatment. The primary cause of cancer morbidity and mortality is cancer metastasis. It is estimated that about 90% of cancer deaths are caused by metastasis [2,3,4,5]. This process is determined as the dissemination of cancer cells from primary tumors to surrounding tissues and to distant organs, which is also known as the invasion-metastasis cascade. One necessary step in distant metastasis is the transport of tumor cells through the blood system, but detailed molecular mechanisms underlying tumor metastasis still remain unclear [6,7]. Detached cancer cells of solid tumor origin from primary tumor which intravasate into the peripheral blood system and circulate in the body are called circulating tumor cells (CTCs). Only a small number of CTCs are able to evade immune attack and extravagate during the circulation at distant capillary beds and seed the growth of a secondary tumor [8]. Consequently, CTCs play an important role as part of a ‘liquid biopsy’ which can offer important information on prediction of cancer progression and survival after specific treatment without surgery [9,10]. The analysis of CTCs includes characterization, determination and enumeration of CTCs. Comparison studies of enumerating CTCs before and after resection open up the possibility for monitoring therapeutic response. Moreover, the enumeration of CTCs also represents an attractive biomarker for predicting the possibility of tumor recurrence [3]. Furthermore, the number of detected CTCs usually correlates with the progression of cancer disease resulting in further information about tumor burden and recurrence [11,12,13]. Additionally, cultivation of isolated patients-derived CTCs can be used for drug resistance analyses and also for the development of personalized anti-cancer agents (Figure 1) [11,14].

During the early stages of tumorigenesis the determination of the existence of CTCs in blood samples of patients is a significant biomarker for early cancer detection [15]. Moreover, CTCs have been detected in many cancer types including breast [16], colon [17], lung [18], melanoma [2], ovarian [19] and prostate cancers [20]. Nevertheless, because of CTCs rarity and their property to move as individual cells or as multi-cellular clumps, their capture and detection are extremely challenging. For example, in 1 mL blood sample of an early stage cancer patient can be detected approximately five billion red blood cells, ten million white blood cells and as few as one CTC [21,22]. Because of biological and molecular changes of CTCs during the epithelial-to-mesenchymal transition (EMT), the circulating cell population is heterogenic and requires the ability to handle a very small number of cells for efficient isolation methods [23].

Over the last few decades, nanoscale materials have been used in a wide range of areas such as electronics, energy conversion, catalysis, storage and medicine. The variety of these advanced nanoscale materials includes metal, metal oxide, semiconductor, polymeric NMs and microbubbles (MBs) [21,24]. Excellent contributions to clinical medicine were made by NMs since they possess some attractive properties related to their size, shape and surface characteristics [25]. Due to the nanoscale effect, NMs have surpassing structural and functional properties that are typically different from either bulk materials or discrete molecules. Although CTCs were already discovered in 1869 by the Australian researcher Thomas Ashworth, only during the last two decades a large number of important advancements have been made in the field of CTC isolation and detection techniques [26]. In recent years, nanomedicine (i.e., here the use of NMs for CTC detection and isolation) has been playing an increasingly important role in CTC detection and more than 100 companies are providing CTC related products and services [27]. The specificity of CTC recognition could be significantly improved by conjugation of NMs with targeting ligands. It has been shown that functionalized substrates and captured CTCs exhibit improved ligand-antigen binding. Nanostructured substrates demonstrate enhanced local topographic interactions that lead to enhanced cell capture affinity. NMs can also be used as drug delivery systems for CTC-targeted drug delivery and cancer treatment [28]. Moreover, NMs have a large surface-to-volume ratio that endows them with a high cellular binding affinity in the complex blood matrix. Additionally, ligand coating of nanostructures can be prepared with much higher density improving binding affinity in comparison to micro- and macrostructures. A manipulation of NMs gives them the ability of multiplexed detection and targeting, which are crucial to approach the heterogeneous problem of CTCs [21]. Furthermore, the use of microfluidic chips as cost-effective, miniaturized and efficiency improved applications for the enrichment and detection of CTCs obtain better performance with nanostructured substrates. When targeted MBs are used, CTCs can readily be separated by simple flotation or gentle centrifugation, preserving cell viability for culture and also providing theranostic capacities.

In this review, we will provide an overview of current CTC enrichment strategies and clarify the relationship between CTCs and tumor metastasis. We will discuss the interactions of nanoparticles (NPs) and MBs with biomolecules such as proteins in biological media, and what consequences this may have on detection and isolation strategies. Some CTC detection and analysis methods will briefly be discussed as a guide for the development of potential clinical diagnostic platforms. Literature on in vitro NPs-based CTCs enrichment systems which have drawn extensive attention due to their clinical potential will also be elaborated in this review. Besides our focus on the “nano”, we will also elucidate the “micro” including complementary microfluidic and lab-on-a-chip technologies for simultaneous CTCs enrichment and analysis. Last but not least, we will summarize the research progress of the development of robust nanosystems and MBs for CTC-targeted cancer therapy.

## 2. The Metastatic Process

As previously mentioned, metastasis is a multi-step process including the spread of cancer cells from primary tumor to distant organs by intravasation into the circulating system. These cells are often called circulating tumor stem cells due to their stem-like properties [29]. CTCs are involved in the process of EMT during early steps of the metastatic cascade [30]. This process is involved in breaking up the cell-to-cell contact by downregulation of various cell adhesion molecules (for example E-cadherin), or epithelial antigens (like epithelial cell adhesion molecule, EpCAM). Due to specific signaling molecules (*Wnt/β-catenin*, *FGF* or *TGFβ1/BMP*) EMT induces cell migration and development of mesenchymal-like cells [31]. During EMT CTCs detach from primary tumor, loose their epithelial character by downregulation of EpCAM, infiltrate the blood circulation system and migrate into distant site of future metastasis [7,32]. At distant sites, CTCs interact with the local microenvironment which leads to its adaption via developmental and self-renewal signaling pathways, like Hh, Wnt and Notch. These signaling pathways are responsible for the proliferation and finally for the forming of metastases [33]. Subsequently, CTCs have to recover their epithelial characteristic to resettle in the target organ. This process is called mesenchymal-to-epithelial transition (MET) and is a reverse process of EMT [34]. The process of MET at distant sites is not fully understood and it is also not known how many factors are responsible for MET activation. Banyard et al. demonstrated evidence for spontaneous MET process in an in vivo model [35]. This researcher group selected and expanded metastatic cancer cells that survived in the lymph node microenvironment of mice bearing human prostate tumors. The progression of lymphatic cancer cells demonstrated the existence of epithelial like cells as a result of MET. There are also evidences for MET activation after switching off EMT transcription factors, such as Twist 1, and silencing the EMT inducer Prrx1 to prevent further EMT and allow CTCs to migrate into distant sites of future metastasis [35,36,37,38].

**Figure 2:**
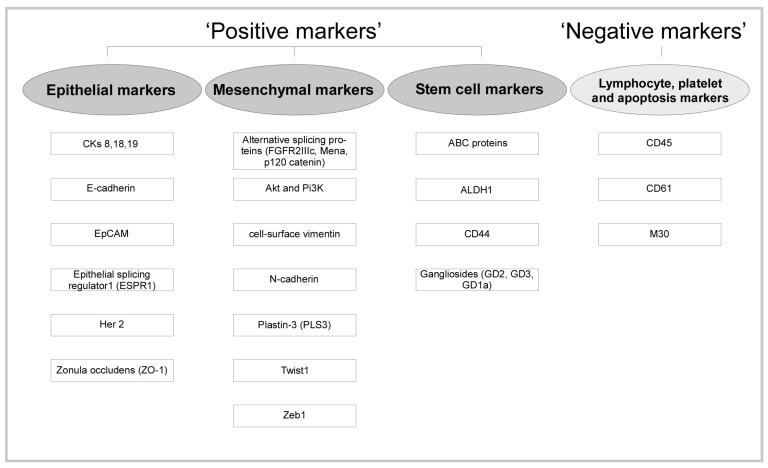
Illustration of CTCs detection specific key biomarkers.

The process of EMT is important for the initiation of a stem cell phenotype which displays some characteristics. During the metastasizing process developed characteristics such as high invasiveness, self-renewal ability and resistance to apoptosis and therapy, are used as biomarkers for detection and isolation of CTCs [39,40]. Specific biomarkers are essential for most biological detection methods. Cancer biomarkers are the measurable molecular changes between normal and cancerous tissues of patients. Each cancer type has specific pathological evolution and molecular characteristics. Consequently, for further applications in CTC capture and isolation the identification of these biomarkers is crucial [41,42]. For example, CTCs are commonly described to express epithelial markers like EpCAM and cytokeratins (CKs), and to be nucleated (identified by staining with a nuclear dye such as DAPI, 4′, 6-diamidino-2-phenylindole). Moreover, CTCs do not express cell surface marker CD45 which is specific for white blood cells [43,44,45,46]. In summary, it can be declared that positive results in CTC specific detection can be obtained by using a variety of epithelial-, mesenchymal-, and stem cell markers. Additionally, in order to determine and eliminate debatable cells ‘negative markers’ could be used for CTC detection. These markers include for example platelet marker CD61, CD45 and apoptosis marker M30 [43,44,45,46]. Some of these key biomarkers are illustrated in Figure 2.

## 3. Use of Nanomaterials in Tumor Detection and Isolation

A high level of sensitivity as well as specificity due to the extreme rarity and heterogeneous phenotype of CTCs in the blood system are necessary for an effective capture and accurate identification of CTCs [28]. Current CTC enrichment methods are based on either biological features (cell surface protein expression, invasive capacity, and viability) or physical properties (size, density, deformability and electrical charge) [47]. Commonly used isolation methods based on physical properties include membrane filtration, flotation, density gradient centrifugation and microchip-based capture platforms. These methods are fast, simple and label-free, but unfortunately less specific. Accordingly, ‘physical-methods’ are usually combined with the antibody-labeled biological method. Examples for biological characteristics-dependent isolation methods include immunomagnetic separation, buoyancy-based separation, substrate- and microchip-based capture platforms [21].

As mentioned, a high level of specificity and sensitivity is important for capture and identification of CTCs. NMs could largely improve the sensitivity and efficiency of CTCs enrichment, isolation and detection. Correspondingly, the unique properties of NMs can be used to accelerate detection and overcome some limitations in CTC detection [28]. It is necessary to understand the interaction of NPs with cells, tissues and organisms in detail to reach safe application of NMs as diagnostic devices in cancer therapy [48].

To understand changes of NMs in complex physiological or natural environments an extensive understanding about the behavioral and physico-chemical properties of NMs is urgently needed [1]. Due to a high surface-to-volume ratio, NPs interact with (bio)-molecules upon contact with biological and abiotic environment and form the so-called (bio)-molecular corona. In complex biological environments including simple and higher organisms this (bio)-molecular corona is formed spontaneously like an adsorption layer on the NP. Additionally, this adsorption layer plays an important role in the interaction of NPs with organisms and control of their physiological responses. The biomolecular adsorption is mediated by different properties of NPs, including composition, shape, size, surface charge and surface functionalization [49,50,51,52]. The protein absorption to NP surfaces is known as the ‘Vroman effect’ and thus was postulated in the pioneering work by Vroman [53]. This effect is described as dynamic change of protein corona composition by adsorption and desorption. This means that in a blood sample containing thousands of different proteins, abundant proteins will be desorbed from NP surface and replaced by rare ones with a higher affinity which leads to a constant level of adsorbed proteins [52,54,55].

In 2007, for the first time the term ‘protein corona’ was introduced to the NP community by Cedervall [54]. The term ‘hard protein corona’ was described as a strong bound layer of biomolecules, representing an analytically approachable protein/biomolecule signature of NP in a determined environment [1,49,55]. Some models additionally describe a ‘soft protein corona’ around the ‘hard protein corona’ which is described as a rapidly exchanging and highly complex biomolecule layer without direct contact to the NPs [1,49,54,56,57]. However, the presence of this ‘soft protein corona’ (also called ‘soft corona cloud’) and its importance at the nano-bio interface are not yet fully affirmed. Moreover, in the context of biology and medicine unspecific ligand-receptor interactions have been discussed and no differences between ‘soft’ and ‘hard’ ligand-receptor interactions were made. Therefore, it is recommended to term the analytically approachable NP-protein complex as ‘protein corona’, because the terming ‘soft’ versus ‘hard’ corona does not take into account all types of coronas and does not assist in resolving pressing scientific questions [1].

### 3.1. Magnetic Nanoparticle-Based System

Magnetic separation using magnetic NPs (MNPs) is principally used for the isolation of CTCs. Assembled in an organic or inorganic matrix, dispersed antibody-labelled MNPs or MNPs clusters are bound to CTCs. Due to this composition, cells can be separated via an external magnetic field [21,22]. During the presence of an external magnetic field a magnetic moment is exhibited by the most commonly used MNPs such as cobalt, chromium, iron and also their oxides [58]. The magnetic response of iron oxide MNPs can be ferromagnetic or superparamagnetic depending on the particle shape and size. Moreover, this type MNPs presents chemically stable and biocompatible features [59]. In comparison to iron oxide MNPs, ferromagnetic NPs have a remnant magnetization after removal of the external magnetic field. Moreover, ferromagnetic NPs demonstrate poor stability leading to aggregation in aqueous media so that these particles are not used for cell isolation. Consequently, superparamagnetic NPs (SMNPs) or clusters composed of SMNPs are suitable for cell isolation because of thermal fluctuations [21,22,60]. Additionally, the surface of SMNPs is often modified by coating or grafting with surfactants, polymers, (polyethylene glycol-PEG), polypeptides or hydrophilic inorganic materials (silica and gold) [22].

**Figure 3:**
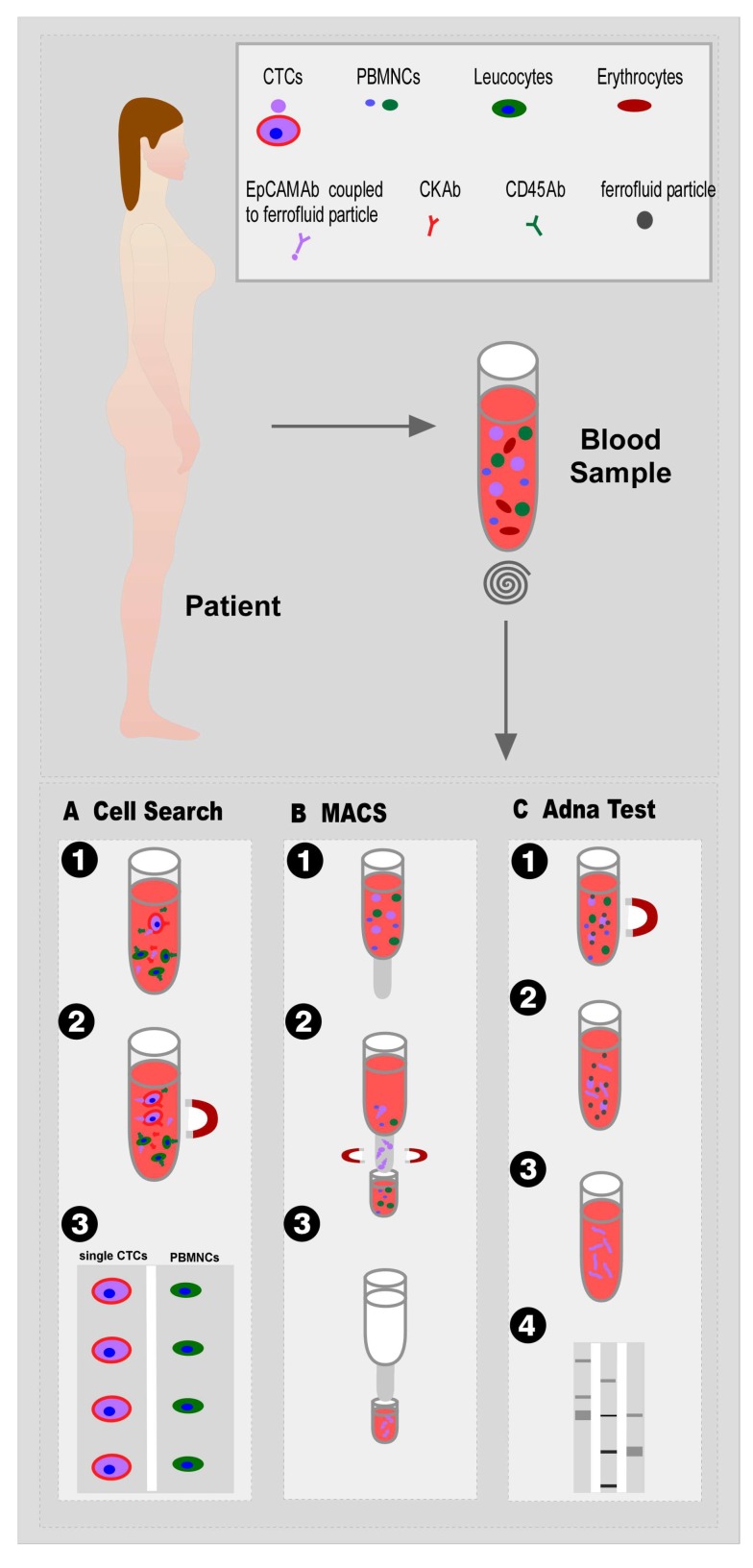
Illustration of CTC isolation methods by magnetic separation using MNPs: (**A**) The *CellSearch* system includes the enrichment of CTCs with ferrofluid particles linked with anti-EpCAM antibodies, magnetic separation of labeled cells and evaluation by immunofluorescent staining. (**B**) The principle of magnetic activated cell sorting (MACS) by using superparamagnetic Fe NPs within a magnetized steel wool column. (**C**) The process of AdnaTest describes the immunomagnetic enrichment of CTCs via epithelial and tumor-specific antigens. Potential CTCs are separated from peripheral blood mononuclear cells (PBMCs) and lysed in order to analyze the CTC gene expression via multiplex PCR.

The most commonly used magnetic system for CTCs isolation is the ‘Food and Drug Administration’ (FDA)-approved Cell Search system (Menarini Silicon Biosystems Inc, Huntington Valley, PA, USA) which is considered to be the gold standard. This system enriches CTCs using iron NPs (ferrofluid particles) linked with anti-EpCAM antibodies [61] (Figure 3A). The CellSearch system is primarily designed for the enumeration of CTCs with an epithelial origin expressing EpCAM and keratin. Due to the proportional correlation of the magnetic force and the number of bound NPs [62], cells can be selectively enriched by making use of the fact that NPs bound cells are isolated faster than free NPs in a solution under the same external permanent magnet field. This process is separated into two steps and also two different instruments: Autoprep is responsible for CTC capture and immunostaining, and CellTracks Analyzer evaluates the immunofluorescent-stained cells by a semi-automated fluorescence microscope. Further immunofluorescence staining with anti-keratin and anti-CD45 can increase the specificity of selected cells [58,63]. Although CellSearch represents a clinically validated method for CTC isolation, this system has to overcome large limitations including the dependence on cells expressing EpCAM and the fact that only a very small proportion of CTCs in the blood sample of a patient can be detected in a limited interval of time. The process of EMT and the accompanied downregulation of epithelial markers like EpCAM have already been discussed above [58].

Schüling et al. demonstrated aptamers as a suitable alternative to antibodies for whole cell detection with many advantages. High binding specificity is one of the key advantages of aptamer used applications. Despite comparable affinities to antibodies, aptamers present a limited affinity to negatively charged targets. Unfortunately, developed aptamer-based lateral flow assays are not commercially available at the moment because of missing integration in new nano-sized technologies [64].

The magnetic activated cell sorting (MACS, Miltenyi Biotec, Bergisch-Gladbach, Germany) represents a variation of the magnetic isolation method. MACS uses superparamagnetic Fe NPs combined with a magnetized steel wool column as a special feature in comparison to another magnetic-based isolation system. Cells can be eluted from the column by removing the column from the external magnetic field (Figure 3B). By using a combination of magnetic beads coupled with various antibodies and also the possibility of labeling cells with fluorescent antibodies, this technique describes a large advantage due to a direct enrichment and evaluation of captured cells without further detaching or staining procedures [65].

Another method using more than one antibody for the magnetic enrichment of CTCs is the AdnaTest (AdnaGen AG, Langenhagen, Germany). AdnaTest allows the immunomagnetic enrichment of CTCs via epithelial and tumor-specific antigens (Figure 3C) by making use of different magnetic microbeads, such as the superparamagnetic DynaBeads. This mixture of magnetic beads is simultaneously conjugated to antibodies against EpCAM and tumor-associated antigens for labeling of CTCs in peripheral blood. Next, labeled cells are lysed, mRNA is extracted from captured cells and transcribed into cDNA. The analysis of the CTC gene expression can be made by a multiplex polymerase chain reaction (PCR) [66,67]. In comparison to CellSearch, AdnaTest exhibits improved enrichment efficiency due to the usage of two antibodies and the size of magnetic particles.

These three methods represent positive selection strategies for the specific isolation of CTCs out of a bulk of other cells. One large limitation of positive CTC selection is the described necessity of the expression of targeted markers on the surface of cells. A possible solution to overcome this hurdle is the use of negative depletion strategies with magnetic beads. For negative depletion a two-step procedure was suggested including lysis of red blood cells and removing white blood cells by labeling with CD45-specific MNPs. In summary, it remains a great challenge to efficiently capture CTCs, reduce the great number of normal blood cells in a sample, and protect rare CTCs from damage during lysis and different washing steps [22].

For implementation of standardized CTC detection methods in daily clinical routine it is indispensable to compare different methods and determine the efficiency of the technology. Andreopoulou et al. compared CellSearch system and AdnaTest to evaluate CTC detection in peripheral blood samples of 55 metastatic breast cancer patients (2012). In this study the *CellSearch* system demonstrated 26 of 55 patients as CTC positive in comparison to 29 of 55 patients detected as CTC positive by using AdnaTest. Consequently, the detection efficiency of CTCs in metastatic breast cancer patients of both compared techniques is comparable. However, more studies are urgently needed to compare the CTC detection efficiency of described positive selection methods by using the same biological samples.

### 3.2. Fluorescence-Based Detection by Using Quantum Dots

Fluorescence detection methods take also an important part in leading techniques for CTC detection. For this reason, the use of organic dyes as imaging agents belongs to the standard, although their use is limited by low signal intensity, spectral overlapping, the necessity of multiple light sources to excite different fluorophores in mixed detection and photobleaching [21,68]. Examples for cytometric methods are immunohistochemical staining, flow cytometry and spectroscopic detection. The advantage of cytometric methods is the possibility to further analyze detected cells, if cell lysis is not necessary for former procedures, and to examine the cell morphology, if cells are reported microscopically. Nucleic acid-based methods assess tumor-specific genetic alterations by analyzing whole cells or extracted RNA or DNA by PCR, RT-PCR, and whole-genome amplification. Due to interference caused by the expression of normal cell markers, nucleic acid based methods usually have a low specificity, but a high sensitivity [21,69].

Quantum dots (QDs) are an example of fluorescent NPs with size-dependent fluorescent emission that can be applied in the field of CTC detection. In comparison to fluorescent proteins and traditional dyes, QDs display a high quantum yield, tunable emission wavelengths and long fluorescence duration which can enhance the sensitivity of surface-marker dependent CTC capture [59]. It is also possible to capture heterogeneous CTCs by using simultaneous multicolor labeling of size-dependent QDs [59,70]. Due to their strong and stable fluorescence, QDs-based ex vivo CTC detection is highly relevant for clinical applications. However, the use of QDs for in vivo CTC detection provoked heavy metal toxicity [71].

### 3.3. Gold Nanoparticles

Gold nanoparticles (Au NPs) are another type of NP extensively used for improving the efficiency of CTCs enrichment and capture due to enhanced light absorption and scattering properties. In previous studies a variety of Au NPs have been synthesized exhibiting different shapes such like nanospheres, -rods or -shells. Subsequently, it is possible to functionalize the surface of these NPs with therapeutic agents, targeting moieties and imaging labels. The interaction of Au NPs and CTCs can be monitored by analyzing the protein adsorption processes at the Au NPs surfaces. By using surface plasmon resonance (SPR), the molecular adsorption is demonstrated by a measurable shift. Furthermore, it is possible to measure the binding between Au NPs and CTCs by using photoacoustic signals [22,58,72,73,74].

Based on unique characteristics, such as high sensitivity, flexibility and throughput, Au NPs are broadly applicable in the field of imaging and diagnostics. One example for an in vivo application is CTCs targeting by injection of Au NPs into the blood stream. This allows real-time, in situ monitoring of CTCs without blood sampling, sample preparation and the following CTCs isolation steps. Furthermore, this application enables the phagocytic clearance of CTCs upon binding [22,58]. Besides these advantages, the method also exhibits some disadvantages due to the particular conditions in the blood system, like high shear stress or immune response. Similar to other CTC targeting methods, injecting Au NPs into the blood may produce false positive results [75]. PEGylation of Au NPs is a commonly used strategy to overcome some of these issues resulting in an extended circulation time and decrease of non-specific binding [76,77].

Modified Au NPs with CTC-specific ligands can be used for direct binding and separation of CTCs from patient blood as an ex vivo approach [71]. This CTC detection method demonstrates two advantages: first, it protects patients from the potential toxicity of labeled NM for CTC-capturing and secondary, it enables cultivation and analysis of the isolated cells. Furthermore, it is possible to bind and enumerate CTCs label-free by immobilization of Au NPs on a nanostructured surface. For example, a thiolated ligand-exchange reaction with Au NPs on a herringbone chip (NP-^HB^CTC-Chip) was used to isolate and release cancer cells from whole blood by Park et al. Antibody-coated NPs were chemically and directly assembled onto the ^HB^CTC-Chip. This application has several advantages in comparison to antibodies coupled on flat silicon oxide surfaces: (1) increasing the available surface area to improve specific interaction of cancer cells with antibodies; (2) release of cancer cells from the surface by disrupting the metal-thiol interaction; (3) usage of released cancer cells for ex vivo cell culture and further molecular analysis; and (4) the optimization of this method for application in complex surface topographies without additional changes in the process by using chemically self-assembled monolayers [22,78].

### 3.4. Graphene and Carbon Nanotubes

Graphene is arranged in a two-dimensional layer of sp^2^ hybridized carbon atoms ordered in a honeycomb network. Additionally, it is the basic structural block of other allotropes such as graphite and carbon nanotubes (CNTs). Unique chemical and physical properties of graphene and graphene oxide (GO) include strong mechanical strength, high surface area, high intrinsic mobility and great thermal conductivity with optical transmittance and electrical conductivity [58,79,80]. The chemical response results in a charge transfer between graphene and adsorbed molecules. GO can be functionalized through PEG-based chemistry and GO size is controllable by sonication time and filtration [81,82]. Moreover, graphene and GO have been used for electrical CTC detection due to the excellent electromagnetic detection of small biomolecules [83] and found their application in biological and medical research by using optical transparency for imaging [84]. The application of a GO chip for sensitive CTC capture was achieved by self-assembled GO nanosheets on a gold-patterned silicon surface via a positively charged intercalating agent and functionalization with PEG [85]. Yoon et al. spiked cells of different cancer cell lines into buffer or blood samples and flowed through a GO chip. Spiked cells were captured due to the usage of anti-EpCAM antibody for substrate functionalization by cross-linker and biotin-avidin linker chemistry. Blood samples from patients with breast, pancreatic and early lung cancer were cultivated on the gold-patterned surface with GO sheets with a capture efficiency of 2–23 CTCs/mL [83].

Furthermore, Wu et al. established an electrochemical protocol for the measurement of two tumor specific markers, such as anti-EpCAM and anti-GPC3, on a captured tumor cell surface by application of a GO film-modified glass carbon electrode [86]. This method allows the marker-dependent capture of tumor cells and enumeration of captured cells by square-wave voltammetry. It was also possible to use detected cells for fluorescent imaging [80,86]. The cultivation of captured CTCs opens the possibility for further applications and analysis [86].

The above-mentioned CNTs demonstrate remarkable electrical, mechanical and physico-chemical properties and are composed of graphitic hollow filaments of alterable lengths reaching up to several hundred micrometers. CNTs are known as two types: single-walled (SWCNTs) that comprise a single cylindrical sheet of graphene and double-/multi-walled (MWCNTs) that are composed of several concentric, coaxial, rolled up graphene sheets. The size of CNTs differs with a diameter typically ranging from 0.4 to 3 nm for SWCNT and from 2 to 200 nm for MWCNT [87]. CNTs are synthesized by chemical vapor deposition [87,88]. Due to electronic properties, the conductance of CNTs can be detected by electron current signals and is depending on chemical binding and mechanical deformations. Carbon nanofibers (CNFs) are also included to the group of CNTs. CNFs defend a less perfect graphene sheet arrangement featuring layers of graphene nanocones, the so called ‘cups’ and usually denote ‘stacked-cup carbon nanotubes’ [89].

First experiments on CTC detection in blood were published by Shao et al. [90] Binding of breast cancer cells to functionalized SWCNTs lead to a measurable decrease of conductivity. This assay contains a sensing area that is able to detect potential CTCs with low protein expression. This application presents the advantage of using samples without enrichment steps for direct cancer cell testing on the one hand, and on the other the challenge of a very small volume of analyzed patient blood (<10 µL) bearing the risk of missing CTCs. There is also the possibility of CTC counting difficulties because the signal is determined by a single cell reaching the space between the electrodes. Another example demonstrates the application of MWCNTs on a sensitive CNT-based biosensor for detection of CTCs from whole blood samples based on binding of anti-EpCAM antibodies to cancer cells and resulting in an increased electron transfer resistance. The detection of cells was demonstrated as an electrical response which was proportional to the concentration of cancer cells [59,91].

## 4. *Nano Meets Micro*—Micro-Mized Tools for Tumor Research

Beside the above described nano-sized systems for CTC detection and isolation, there are also techniques extending to the micro scale. Since these methods are widely used in combination with NMs in order to complement and advance nanomedical applications, we also discuss selected examples in the following sections.

### 4.1. Microfibricated Filters

Membrane microfilter devices are a suitable tool for separation of CTCs from whole blood samples by cell size exclusion [13,46,92,93]. Whereas CTCs can vary in their size and shape, the typical smaller dimensions of blood cells are 5–9 μm for erythrocytes, 10–15 μm for granulocytes, 7–18 μm for lymphocytes and 12–20 μm for monocytes [93]. The size exclusion approach is composed of a parylene-based membrane microfilter device including two parylene membrane layers and a photolithography-defined gap to minimize stress. This is the reason why isolated cells are viable and can be used for further molecular analysis [92,93]. The possibility of label-free isolation of CTCs is a large advantage of this technology. However the sensitivity to cell-size in a blood sample can lead to the risk of losing CTCs which are smaller than the filter pores because of their size and shape heterogeneity [46]. As an example, a parylene-based membrane microfilter device with integrated electrodes containing 11 µm diameter circular pores was used to isolate cancer cells. These cells were pre-stained with hematoxylin and spiked into a blood sample. This cell suspension was loaded into a syringe and dispensed to pass through the filter. The flow-through was collected by the bottom syringe. After the isolation process, immunostaining was used to determine potential CTCs from other cells on the filter. The recovery rate of the membrane filters was evaluated by hemocytometer using the hematoxylin staining of spiked cancer cells and resulted in 89.0 ± 9.5% recovery from blood [93].

### 4.2. Microbubbles in Diagnosis and CTC Detection

MBs are gas-filled, echogenic bubbles with a diameter typically comprised between 0.5 and 10 µm commonly used as contrast agents (CAs) in medical imaging and as carriers for targeted drug delivery, recently also gaining attention in the field of cancer diagnosis and treatment [94,95]. They consist of a low solubility complex gas, such as a perfluorocarbon (PFC) gas, surrounded by an external shell generally composed of phospholipids. A mixture of lipids in chloroform is homogenized by sonication in the presence of gas. PFC is especially suitable due to its low solubility in water, which is necessary for maintaining MB stability in the aqueous phase [96]. The bubble size is predominately determined by the solubility degree and partial pressure of the gas [96,97].

The MBs most investigated have soft shells made of phospholipids, sometimes of denaturated albumin, and contain a fluorocarbon (FC) as or among their inner gas phase component(s). Standard shell phospholipids include dimyristoylphosphatidylcholine (DMPC) and dipalmitoyl- phosphatidylcholine (DPPC) [96,98]. The longer chain distearoylphosphatidylcholine (DSPC) forms semi-crystalline liquid-condensed monolayers that confer additional shell rigidity and stability. Pegylated lipids can provide stealthiness. The lipids and PEG chains can be fitted with a wide range of ligands. Polymeric shells can provide some additional stability but their response to UlS waves is usually dampened. The biologically inert inner FC gas, by considerably increasing lipid-coated bubble stability, made the development of commercial CAs a reality. The FC stabilizes MBs by drastically reducing the solubility of the inner gas in the continuous aqueous phase, by osmotically stabilizing the gas phase, and by providing co-surfactant activity with the phospholipids [96,99]. 

**Figure 4:**
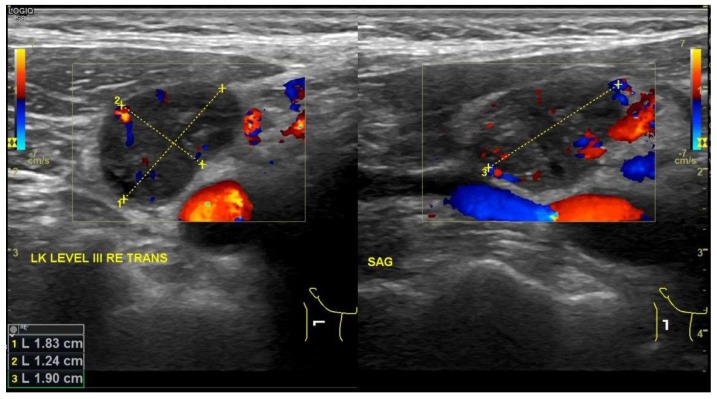
Ultrasound images of right transverse lymph node level III. Lymph node demonstrates malignant characteristics: axes are larger than 1 cm (left image), round shape with necrotic areas (right image).

Furthermore, MBs were developed as CAs for conventional ultrasound (UlS)-imaging diagnostics and have been used for daily clinical practice for more than 20 years (Figure 4). Given their size and rheology comparable to red blood cells, MBs freely circulate through vessels and capillaries, with an average lifetime of 5 min. UlS triggers MBs resonance, resulting in detectable harmonic signals. Molecular imaging can be performed with MBs containing targeting ligands on the bubble-shell. During systemic circulation, targeted MBs progressively accumulate in the regions expressing the targeted molecules, defining areas of bright signals on UlS pictures [100,101]. As a consequence, the use of microbubbles for UlS-imaging can enhance their quality by precisely defining the region of targeted MBs accumulation. For example, MBs can attach to the vascular endothelium via a specific ligand-receptor bond, so that pathophysiological processes (like inflammation, angiogenesis, thrombosis, and tumors) can be imaged [101]. Multiple MBs fitted with targeting devices have been reported that are able to seek inflammation sites, myocardial ischemia, ischemia-reperfusion injury, ischemic memory, atherosclerotic plaques, thrombi, and angiogenesis in malignant solid tumors for the purpose of molecular imaging and focused therapy [102]. A perfluorobutane/lipid MB (BR55, Bracco, Milano, Italy) fitted with a heterodimeric peptide that has affinity for the vascular endothelial growth factor receptor type 2 (VEGFR2), a molecule expressed in neoangiogenesis, and hence, can help detect angiogenesis, has recently (2016) been licensed by the FDA for molecular imaging and characterization of liver masses and as intravesical contrast agent for voiding cystoureterography in children. This agent and other targeted MBs are also being investigated for detection of prostate, ovary and breast cancers [103,104,105].

Effective detection and isolation of CTC cells require high specificity and sensitivity, and simple and cost-effective technology. Targeted FC MBs are among the devices that are being investigated for CTC detection. They are easy to produce and collect cost-effectively. They can readily be fitted with multiple ligands, antibodies, and other markers that can recognize CTCs [102,103,106,107,108,109]. Due to their low density and buoyancy in aqueous media, they can easily be separated from aqueous media by flotation or gentle centrifugation. Once harvested, the isolated CTCs can be characterized, which can help determine treatment. CTCs may also be cultivated for personalized/precise drug testing. Besides their use for CTC isolation, MBs seem to enable CTC-targeted cancer drug delivery [102] (see Section 5.4) and may serve as carriers for drugs, genes and various markers, to overcome pathophysiological barriers, such as the blood brain barrier. Notably, MBs are furthermore already used for the delivery of energy, thus enabling techniques such as tissue ablation, sonothrombolysis or embolotherapy [102,110,111,112]. These approaches are technologically manageable, efficient and do not require any complex equipment. Their stability is limited, however, particular in biological fluids. But progress in formulation and preparation procedures has provided MBs sufficiently stable for efficient patient examination and for analytical procedures. MBs are extremely sensitive to UlS waves and can be imaged, monitored and manipulated by UlS [96,106]. Other imaging modalities, including ^19^F-MRI can often be applied [113,114].

The use of MBs for CTC detection is very promising in theory but remains limited so far. Indeed, only one procedure using MBs to assist CTC separation from biological fluids has been reported [108,115]. This method is called flotation separation. The potential for targeted FC MBs to selectively bind and separate by buoyancy certain populations of circulating blood cells, initially erythrocytes and B-lymphoma cells, has been established [115]. This buoyancy-separation principle originally allowed for the isolation of CD4+ T lymphocytes from peripheral blood, following mixture with glass MBs [116]. In oncology, the capture of tumor cells has been demonstrated in solutions, blood or large-volume buffy coats [108,115]. Pancreatic tumor cells were captured within 15–30 min of incubation with functionalized MBs. The MB binding efficiency to human lung and mouse breast carcinoma into BSA/PBS or blood (around 90%) was comparable to that of commercial anti-EpCAM-coated magnetic beads (DynaBeads), ranging between 60–90% [117]. The EpCAM-targeted MBs can bind efficiently (85%) and rapidly (within 15 min) to various epithelial tumor cells suspended in cell medium [109]. In plasma-depleted blood, such MBs isolated tumor cells at high (105–106 cells/mL) and low (10–20 cells/mL) concentrations of tumor cells (mouse breast 4T1, human prostate PC-3 and pancreatic cancer BxPC-3 cells). However, in whole blood, MBs presented decreased stability, possibly due to gas mixing and exchange. Further development of the method led then to the design of blood-stable MBs for isolation of breast tumor cells [102]. Parallel studies on the lipid shell functionalization with anti-human EpCAM or EGFR antibodies demonstrated different preferential binding abilities to several breast tumor cell lines with distinct marker expression profiles, and culminated in the production of multi-targeted anti-human EpCAM/EGFR MBs recognizing all cell lines with over 95% efficiency [102]. Fast (30 min) and efficient (70–90%) recovery of CTCs was achieved in human blood. In patients with metastatic breast cancer, these MBs allowed for the isolation of CTCs, cell clusters and tumor-derived CK+/CD45- microparticles. Also, albumin-based MBs have been used for buoyancy-activated cell sorting, which showed inherent advantages (such as stability and simplicity of formulation) with respect to lipid-based ones [118]. In albumin-systems, the most common way for antibody conjugation is based on the non-covalent incorporation into the albumin shell of avidin linkers as anchor sites for biotinylated antibodies. Nevertheless, to strengthen the antibody conjugation, biotin can be first connected by a covalent amide bond to albumin, followed by incubation with avidin and biotinylated antibodies. These biotin-MBs targeted against CD44 receptors efficiently recognized luminal breast cancer cells in PBS, and were able to separate them from the CD44- basal-like breast cancer subset with higher sorting purity than other control MBs [118]. These results contribute to establishing that targeted MBs are an effective new approach to liquid biopsy [119]. As compared to other methods (adherence, absorbance, particle size, density gradient, dielectric properties, chemo-resistance), the antigen-antibody recognition provides precise sorting. The two major sorting tools, being fluorescence activated cell sorting (FACS) and MACS, use expensive and large instruments, long processing time, and magnetic forces that may damage some types of cells [120,121,122]. Similarly, microfluidic approaches exert substantial shear stresses thus risking cell damage [123]. Instead, buoyant MBs isolate specific cells with molecular precision in a simple and safe way, given that the shear stress originated from a rising bubble and the tension from the buoyancy force are both far below the threshold for cell damage [124]. Moreover, whereas the assays where nano- or micron-sized immunomagnetic beads capture the CTCs suffer from some other limitations (such as non-specific carryover, relatively long processing time and contamination with leukocytes) [125,126,127], the MB-assisted cell isolation emerges as a promising method for rapid and accurate collection of exfoliated tumor cells in a variety of pathological samples (e.g., blood, bone marrow, urine). Finally, besides being cost-efficient and scalable, the flotation separation technology presents also wide horizons of optimization for biological use, considering the large versatility of MB surface functionalization.

MBs can also deliver therapeutic agents, and hence, exhibit extended theranostic potential. Remarkably, it was found that exposure to a supernatant FC gas can significantly enhance the adsorption and retention of a large variety of molecules, including lipids, proteins, surfactants, poloxamers, fluorinated drugs and hypoxia biomarkers at the surface of lipid-shelled MBs. The same phenomenon has been observed with diverse NPs, such as magnetic iron oxide, cerium oxide or nanodiamonds. The effect is particularly marked for fluorinated molecules and particles.

Multiple hybrid MBs are also being developed that carry NPs enclosed in or attached to their shell, including superparamagnetic iron oxides [128,129], QDs [130], gold clusters or nanorods [102,131,132], GO sheets [133], cerium oxide NPs [134], liposomes [135]. Small and stable MBs decorated with dendronized iron oxide magnetic NPs were obtained that are stabilized by fluorine-fluorine interactions between the internal FC gas and the fluorinated terminal end-group of the oligo(ethylene glycol)-based dendrons [136].

### 4.3. Microfluidic Lab-on-a-Chip Devices

**Figure 5:**
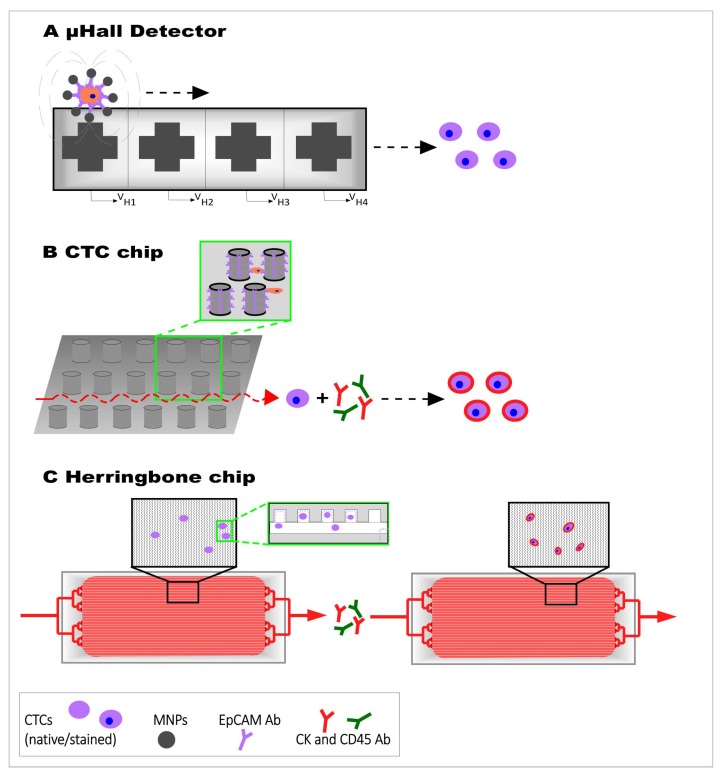
Design of microfluidic chips for CTC detection. Whole blood sample is pushed through the surface of the chip. (**A**) Cells are MNPs-immunolabeled and can be detected via monitoring the magnetic moments of cells in-flow on µHall detection chip. (**B**) CTC chip is coated with a CTC-specific antibody, such as EpCAM, and contains Ab-coated microposts. This system is also used in herringbone chip that contains Ab-coated microchannels (**C**). Captured cells are stained for CK, CD45 and DAPI for identification and enumeration.

A new development in the field of CTC enrichment and detection includes microfluidic lab-on-a-chip devices with immense advantages including cost-effectiveness, miniaturization, and the improvement of efficiency since it could be integrated into other techniques [14,137]. Consequently, isolation and analysis of CTCs on one chip can improve the number of catched CTCs by avoiding loss of rare cells during the experimental steps of sorting, enumeration and analysis [138]. Current microchip platforms are based on magnetic force, affinity, size or other physical properties and are separated into two types of microfluidic devices for CTC detection [139]. The first type of microfluidic devices includes the immunomagnetic-based method for CTC detection (e.g., CTC chip) and the second type represents the method of antibody-labeling combined with physical isolation (Figure 5), which can consist of different materials like silicon, glass or thermopolymer.

### 4.4. Immunomagnetic-Based Method and ‘Micro-Hall Detector’

Immunomagnetic-based CTC chip separation is performed by using the advantages of two combined techniques: the immunomagnetic separation and the microfluidic device. The capture efficiency depends on the magnetic strength and drag force under the flow condition. Cells bound to large number of NPs can be captured more efficiently by using both forces [21]. The isolation of cells in microfluidic channels is performed in the presence of a permanent magnetic field which can be located under the bottom of the chip [80,140] or on top of the channel to improve the separation efficiency by inverting the microchannel that results in gravity direction opposite to the magnetic field [141].

The possibility for a fast screening method for CTCs in a blood sample with a miniaturized microfluidic technology is called micro-Hall detector (µHD). The µHD can selectively and sensitively detect a wide range of single cellular biomarkers or multiple biomarkers on individual cells as screening system (Figure 5A). MNPs-immunolabeled cells can be detected via monitoring the magnetic moments of cells in-flow on a single microfluidic chip. There is also an option to use MNPs with different magnetization properties to label different cellular markers. By using the particles’ classifiable magnetization properties, the quantity of each MNP type representing the expression level of a distinct target biomarker in a single cell can be obtained [142].

### 4.5. ‘CTC –Chip’ as Silicon-Based Alternative

Another microfluidic device used for efficient and reproducible isolation of CTCs from the blood of patients with common epithelial tumors is called ‘CTC-chip’ [143]. This microfluidic system is composed of three parts: the CTC-chip etched in silicon, a manifold to enclose the chip, and a pump producing the flow through the capture module (Figure 5B). Additionally, the CTC-chip contains an EpCAM-antibody functionalized array of microposts. The cell capture efficiency can be influenced by two essential parameters: flow speed and shear force. The flow speed is important due to its influence on the duration of cell-micropost contact, whereas the shear force has to be minimized to guarantee a high cell-micropost attachment [143].

### 4.6. Glass/PDMS-Based Chip and ‘Herringsbone-Chip’

After the development of the CTC-chip a modified herringbone CTC capture chip was developed to increase the interaction of flowing cells and anti-EpCAM-functionalized polydimethylsiloxane (PDMS) microchannels through passive mixing [144] (Figure 5C). The ‘herringbone-chip’ has integrated microvortices to disrupt streamlines and increase the capture efficiency. Due to the antibody-antigen interaction, cells tether to the chip and can be stained afterwards. The advantage of this glass chips is the transparency that allows clear imaging by using different types of light microscopy based techniques [80].

### 4.7. Thermoresponsive Polymer-Based CTC Chip

Due to magnificent optical transparency and low costs polymethylmethacrylate (PMMA) is also used for microfluidic CTC capture and analysis. PMMA includes UV exposure generated carboxylic acid groups on the surface to analyze protein concentration, electroless deposition and cancer cell capture [145]. Due to the enhanced surface area for functionalization, the surface area roughness can be additionally increased by high intensity light. The thermal bonding passes through to preserve these microfeatures at low temperature [146]. Accordingly, for CTCs specific capture and enumeration a high-throughput microsampling unit functionalized with anti-EpCAM antibodies and an included conductivity sensor has been developed [80].

## 5. Applications in Nanomedicine

**Figure 6:**
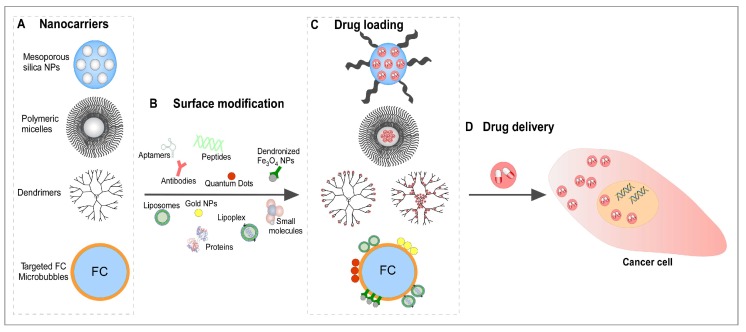
Illustration of drug delivery system of different nanocarriers: (**A**) Unloaded nanocarriers: Mesoporous silica NPs, polymeric micelles, dendrimers and targeted FC microbubbles. (**B**) Surface modification of nanocarriers with cancer cell specific targets (aptamers, antibodies, dendronized Fe_3_O_4_ NPs, gold NPs, liposomes, lipoplex, peptides, proteins, quantum dots, and small molecules). (**C**) Illustration of drug loaded nanocarriers and drug release in cancer cells (**D**).

As fighting tumor metastasis is, besides elimination of the primary tumor, the overarching goal of chemotherapy, therapeutic targeting and specific depletion or destruction of CTCs from blood vessels may be an intriguing strategy for prevention of tumor metastasis. With the emerging possibilities of nanoscale materials, researchers have new tools at hand to design and develop a variety of nanosystems for targeted delivery of therapeutic agents to CTCs hoping to efficiently destroy them and thereby to inhibit tumor metastasis (Figure 6).

### 5.1. Mesoporous Silica Nanoparticles

Mesoporous silica nanoparticles (MSNs) with a pore size from 2 nm to 50 nm show attractive properties, such as a large surface area, mesoporous structure as well as a controllable pore size, ease of surface functionalization and good biocompatibility [147] and have therefore drawn considerable attention especially for drug delivery applications during the past years. For example, nanoplatforms have been developed, which can specifically target colorectal cancer cells via an EpCAM antibody-functionalization representing an in vitro model for CTC targeting in colorectal cancer patients [148]. Furthermore, loading of MSNs with mifepristone allowed the inhibition of lung metastasis in mice. Thus, this proof-of-concept study suggested the MSN-based prevention of metastasis spread by restraining CTC activity [148].

### 5.2. Polymeric Colloidal Particles as Polymeric Micelles

Polymeric colloidal particles can be produced in a size range of several nm and allow for secure encapsulation, adsorption or conjugation of therapeutic agents within their polymeric matrix or their surface [149,150]. Due to their biodegradability and biocompatibility as well as the possibility to combine a broad range of controlled chemical and physical properties by molecular synthesis, polymeric materials remain an important cancer drug delivery system. Linear, globular or branched polymers of different sizes have been used in the past [151,152,153]. Core-shell particles, which are mainly formed using amphiphilic block copolymers, are called “micelles”. Micelles consist of a hydrophobic core to minimize aqueous exposure and a hydrophilic shell to stabilize the core [154]. These properties leave micelle structures attractive for drug delivery applications as they allow the loading with hydrophobic small molecule drugs into their core while a steric protection can be added to the outer “shell” layer. Additionally, hydrophilic drugs including macromolecules like nucleic acids can be included into polymeric NPs by electrostatic attraction or chemical conjugation. Moreover, controlled release of macromolecules from micelles has now been applied in different studies [155,156].

In the design of different drug-loaded nano-systems, a number of materials have been applied and were found to be well suited, albeit showing different advantages depending on the application. Polyamides, poly(amino acids), polyesters and polyacrylamides with thermoplastic aliphatic polyesters such as poly glycolic acid, polylactic acid (PLA) and copolymer poly (lactic co-glycolic acid) (PLGA) are some of the most common examples [157,158,159,160]. Due to its high biodegradability, PLGA is often utilized in biomedical applications [156,161]. Furthermore, PLA and chitosan form polymeric micelles [149] whereas the latter can be applied as transport vehicle for hydrophilic drugs. Similarly, PLGA and PLA exhibit advantageous characteristics such as low toxicity in combination with negative surface charge [162]. Nonspecific side effects of the antitumor agent Doxorubicin (Dox) could be reduced by Dox encapsulation in chitosan NPs tested for the treatment of solid tumors in vivo [163,164].

Deng et al. usd Dox-loaded biodegradable polymeric micelles to target CTCs and finally suppress tumor metastasis [165]. They synthesize monomethyl poly (ethylene glycol)-poly (e-caprolactone) (mPEG-PCL) deblock copolymers to prepare Dox-loaded micelles via a pH-induced self-assembly. Whereas unloaded micelles showed minimal cytotoxicity when incubated with 4T1 cells even at very high concentrations, micelles loaded with Dox induced slightly higher cytotoxicity than Dox alone. Dox micelles were able to inhibit tumor growth, suppress tumor metastasis by killing CTCs and extend the survival rate in transgenic zebrafish as well as a mouse model, by inducing apoptosis and reducing the number of proliferation-positive cells in tumors [165].

In another study, a designed nanoplatform consisting of paclitaxel-loaded PEG-PLA polymeric micelles was successfully applied to achieve dual damaging of the primary tumor as well as CTCs [166]. In a recent study, Gener et al. loaded polymeric micelles with the FDA-approved drug Zileuton™ which has been reported to be a potent inhibitor of cancer stem cells. Interestingly, the authors reported complete eradication of CTCs in the blood stream of an in vivo mouse model and thus, suggested their system to be effective against metastatic spread [167].

### 5.3. Dendrimers

Dendrimers are highly soluble, can be synthesized with a uniformity in size and composition, and have a high number of surface groups. This combination of properties and the ease of functionalizing the surface groups, make them interesting to develop drug development strategies [168]. Employing a novel polyamidoamine dendrimer-based nanoplatform, CTCs could be captured captured and their adhesion to the vascular endothelial layer inhibited [169,170,171,172]. The nanoscale dendrimers therefore made use of two antibodies coated to their surface and targeting membrane markers of human colorectal CTCs (anti-EpCAM and -Slex). Whereas it has been reported, that targeting of EpCAM can directly disturb the adhesion process of CTCs, the Slex (saliva acidifying louis oligosaccharides X) antibody can indirectly interrupt the adhesion between CTCs and endothelial cells via Slex/E-selection interaction [169]. In comparison to their single antibody-coated counterparts, the dual antibody conjugates displayed a remarkably enhanced efficiency and specificity in recognizing and capturing CTCs from a large population of leukocytes or red blood cells *in vitro*, as well as from the blood of patients and mice *in vivo*. Recently, this group developed dual aptamer rings which are conjugated on dendrimers and thus, are able to simultaneously target EpCAM and Her2 biomarkers on CTCs in the presence of millions of normal cells with excellent stability and accuracy [173]. The described study provides new ideas for the design of more powerful and intelligent nanomedicines allowing the prevention of tumor metastasis via suppressing CTCs and blocking their adhesion to blood vessels. Zheng et al. presented a type of barcode particle consisted of spherical colloidal crystal clusters which are surrounded by dendrimer-amplified aptamer probes [174]. A specific aptamer functionalization let the particles interact with specific CTC types and used dendrimers able to amplify the effect of the aptamers. Particles with these capabilities are able to capture, detect and release multiple types of CTCs from clinical samples [174].

### 5.4. Microbubbles in Tumor Treatment

In recent years, MBs have started to be used for therapeutic approaches [175]. Chemotherapeutic agents can be delivered to malignant tissues by combining UlS and MBs, enhancing the in vivo delivery of the drug into the tumor, thereby minimizing the harmful systemic side effects on normal tissues [176]. In this regard, focused UlS in the presence of circulating MBs have been extensively exploited to temporarily open the blood-brain barrier and elicit the passage of chemotherapeutic agents into neural neoplasms. In fact, the local tumor insonation elicits a stable *cavitation* effect, as the UlS energy is transferred to the circulating MBs, letting them to expand and contract cyclically. This oscillation results in damage of the tight junctions and interruption of the contiguous endothelial cell layer [177].

Novel MBs have been developed to directly be loaded with drugs and to release them, thus acting as drug delivery platforms, either in the presence or in the absence of UlS stimulation. Strategies for incorporation of several drugs onto the MB surface have been reviewed [107,178]. The mechanisms of drug release and tumor delivery are different. For instance, the cavitation can cause MB rupture during tumor insonation and drive the drug throughout the capillary wall by delivering a “*ballistic effect*” [179]; alternatively, sonication can induce MB oscillation leading to permeabilization of the contiguous cell membranes, enhancing the entry of a locally released agent into either cancerous or endothelial cells [180,181]. Furthermore, MBs loaded with specific molecules, called *sonosensitizers*, can be used for sonodynamic tumor therapy [175,182]. In sonodynamic therapy, cell cultures or tumors are sonicated by UlS at selected frequency and intensity range (usually around 1.0–2.0 MHz, 0.5–3.0 W.cm^−2^), resulting in phenomena of inertial cavitation. The rapid collapse of bubbles in the liquid milieu determines shock waves producing free radicals and a cascade of molecular events that activate the sonosensitizers, which in turn kill rapidly dividing cancer cells nearby [183]. Conventional sonosensitizers are the same light-sensitive agents developed for photodynamic therapy [184], like hematoporphyrin and its derivatives. When loaded onto MBs [175], their circulation into the tumor vasculature can then be monitored by diagnostic UlS, so that the sonodynamic process can be initiated once detected within the mass, thus creating a therapeutic-diagnostic platform to monitor the treatment effectiveness [185,186,187]. In addition, damaging of the tumor vascularity can also be achieved through MBs (i.e., *antivascular therapy*). Here, MBs act as vascular disrupting agents, whose insonation results into local thermal and cavitation effects leading to the destruction of endothelial cells lining the tumor vessels [188] and necrosis of the neoplastic cells, with a consequent reduction in tumor growth and lengthened survival time. Finally, targeted MBs could be used to selectively eliminate CTCs during their systemic circulation: after intrasystemic injection and CTCs recognition, their insonation might induce local cavitation effects and stimulate either release of chemotherapeutics or cell damage. Moreover, the possibility to combine MBs with other nano-objects (such as liposomes or superparamagnetic NPs) widens the plethora of therapeutic options that one could exploit for CTC killing, including new strategies of drug loading/release and thermotherapy. Nevertheless, even though the principle of the CTC-eliminating MBs could be easily applied, thus far only one study of such kind has been carried out [189]: here, liposome-loaded MBs targeted to N-cadherin (N-cad) could bind to a human melanoma cell line derived from a lymph node metastasis (HMB2 cells). Upon insonation, a model drug (propidium iodide) loaded onto the liposomes was intracellularly delivered to N-cad-expressing cells only. Due to the great potential of MB-mediated CTC depletion strategy and to the urgency in finding effective and portable solutions to hinder cancer relapse caused by metastatic colonization, more efforts in development of such technology are expected in a near future.

## 6. Conclusions and Outlook

In the field of nanomedicine, the application of engineered NMs has assumed an increasing role in early cancer diagnosis and efficient treatment. The analysis of captured CTCs in liquid biopsies from cancer patients provides important information about the biology of cancer micrometastases, and offers a well-tolerated alternative to standard biopsies in the clinical management of carcinoma patients.

Preliminary results of CTCs’ enumeration and analysis obtained by the FDA-approved CellSearch system suggest the possibility of ‘on-line’ monitoring of an ongoing therapy and the drug efficiency. In recent years, a variety of CTC isolation assays have been evolved for supervising a range of distinct tumor types at different disease stages. Due to the extremely rare presence of CTCs in peripheral blood the isolation and detection of CTCs can be very challenging. Consequently, new technologies have to accomplish the challenges of rare cells physical properties including their size, density, deformability and cell shape. Therefore, specificity and sensitivity and cost-effectiveness remain the key issues which upcoming technologies need to address. The development of nanotechnology-based methods for detection and follow-up analysis of CTCs represents a milestone to achieve high capture efficiency, accuracy, and sensitivity. NMs can implement the possibility of a multiplexed targeting because of their possibility to be modified with different targeting ligands to capture, isolate and detect CTC subpopulations.

By summarizing a variety of NM-based enrichment, capture and also detection methods, and by comparing them to complementary micro-sized systems, such as the use of microbubbles, the advantages, but also some disadvantages of nano-sized systems became obvious. Obvious advantages of nanostructured substrates and platforms for the detection and capture of CTCs are better ligand-antigen binding between the functionalized substrate and captured CTCs. Nanostructured substrates demonstrate enhanced local topographic interactions that lead to enhanced cell capture affinity. Additionally, ligand coating of nanostructures can be prepared with much higher density thereby improving binding affinity in comparison to micro- and macrostructures. Moreover, the use of microfluidic chips as cost-effective, miniaturized and efficiency improved applications for the enrichment and detection of CTCs obtain better performance with nanostructured substrates. In contrast, few nanomaterials have made it to clinical trials, or even clinical practice and there is not yet any FDA-approved nanomedical product that markedly improves patient survival or quality of life. The use of NMs in nanomedicine products (e.g., devices and therapeutics) has been quite limited due to their frequent toxic and harmful properties in biological and medical contexts [190]. To however improve therapeutic gain of nanomedicine also in the CTC field, a mechanistic understanding of determinants at nanobio-interfaces is a must.

The benefits of microchip technology and nanotechnology are associated with a combination of NMs with microfluidic devices to optimize the CTC capture methods for further analysis. Targeted lipid-coated FC MBs may ensure rapid and efficient isolation by simple flotation of CTCs from the blood of patients with metastatic cancer, and enable focused drug delivery as well. Besides detection and analysis of CTCs, a huge potential of this technology lies in a CTC-targeted cancer therapy to eliminate tumor cells in the peripheral blood. In conclusion, the further development of nano-sized CTC systems should be not only focused on tumor diagnosis and monitoring, but should also exploit its own potential as anticancer treatment. To benefit from a combination of available technologies, the “nano meets micro” approach seems to be promising to achieve long overdue progress in the field of nanomedicine.
